# The Efficacy of Mesenchymal Stem Cell Therapy in Large Animal Models of Acute Liver Failure: A Meta-Analysis

**DOI:** 10.3390/ijms27073175

**Published:** 2026-03-31

**Authors:** Yuxin Zhang, Yun Yu, Shanwei Yang, Yechao Lu, Xiaoping Pan

**Affiliations:** 1 The Second Clinical Medical College of Zhejiang Chinese Medical University, Hangzhou 310053, China; 202312212601001@zcmu.edu.cn (Y.Z.); 13858034115@163.com (Y.Y.); yangsw716@163.com (S.Y.); yechaolu108@163.com (Y.L.); 2 Department of Internal Medicine, College of Basic Medical Sciences, Zhejiang Chinese Medical University, Hangzhou 310053, China

**Keywords:** mesenchymal stem cells, large animal models, acute liver failure, cell transplantation, meta-analysis, efficacy, safety

## Abstract

Mesenchymal stem cells (MSCs) show therapeutic effects for acute liver failure (ALF), as demonstrated in small animal models of ALF, which showed improved survival and liver function. Nevertheless, small animal models are limited by their simplified immune systems and lower pathophysiological complexity, which prevent them from fully capturing the key features of human ALF. Large animal models offer better physiological similarity; however, the effectiveness of MSC therapy on large animal models of ALF, such as pigs and monkeys, remains unclear. In this study, we performed a meta-analysis to comprehensively evaluate the therapeutic effect and safety of MSC therapy in large animal models of ALF. A comprehensive search was conducted across PubMed/Medline, Web of Science, Embase, and the Cochrane Library for studies published prior to 3 March 2025. Of the 609 identified studies, 13 were included, with the majority showing a low or unclear risk of bias. The results of the meta-analysis indicated that MSC therapy was associated with a higher survival rate and lower levels of alanine aminotransferase (ALT) and aspartate aminotransferase (AST) in large animal models with ALF, compared with the control groups. Subgroup analyses showed efficacy in both pig and monkey models. Furthermore, they showed that bone marrow-derived mesenchymal stem cells and the deep vein transplantation route were each linked to a significantly higher survival rate and to lower ALT and AST levels after treatment in pigs with ALF. Additionally, a dose of (3.0–3.3) × 10^6^/kg was associated with a significantly higher survival rate, as well as a lower AST level after treatment. In summary, the findings suggest MSC therapy is a safe and potential therapeutic option for large animals with ALF, although randomized controlled trials (RCTs) are needed for further validation.

## 1. Introduction

Acute liver failure (ALF) is a life-threatening clinical syndrome characterized by extensive hepatocyte necrosis and the rapid deterioration of liver function. Currently, the most effective treatment is orthotopic liver transplantation (OLT). However, OLT is severely restricted by donor shortages, surgical risks, and the use of immunosuppressants [1,2,3,4]. Although artificial liver support systems can temporarily replace liver function, they may fail to reverse hepatocyte necrosis or severe damage and may not stimulate hepatocyte regeneration. Therefore, there is a need for the urgent development of innovative therapies targeting liver tissue repair and immune modulation [5].

Mesenchymal stem cells (MSCs) have unique biological features that open new avenues for ALF treatment. They not only possess multi-lineage differentiation potential but also release various bioactive factors such as exosomes, growth factors, and anti-inflammatory cytokines. These factors suppress excessive inflammation, promote angiogenesis and hepatocyte regeneration, and reduce oxidative stress damage [6,7,8,9]. Data from numerous rodent studies show that MSC transplantation significantly lowers the serum ALT level, ameliorates liver tissue pathology scores, and increases survival rates in mice with ALF [10,11]. However, the significant differences between rodents and humans in liver anatomy, drug metabolism, and immune responses mean that findings from rodent studies may not directly translate to clinical practice [12]. These limitations hinder the ability of small animal models to accurately predict therapeutic outcomes in humans.

Large animal models offer higher translational potential from basic research to clinical therapy due to their high physiological similarities to humans. Among these, pig models are extensively used for drug-induced liver injury and ALF caused by surgical resection owing to their similarity to humans in their liver anatomy and metabolic features, including cytochrome P450 enzyme profiles [13,14,15]. In addition, non-human primates share most of their genetic makeup with humans and have highly conserved immune systems, enabling them to better reflect the immunomodulatory effects and potential risk of rejection with MSC therapy. Studies using primate ALF models have demonstrated that the peripheral infusion of hUC-MSCs effectively suppresses monocyte activation and cytokine storms, confirming their immunomodulatory effects [16,17]. Using both pig and primate models provides valuable experimental insights that can inform the development of clinical protocols for MSC transplantation in ALF.

In previous studies on MSC transplantation, large animal models of this disease have shown positive therapeutic results. According to Shi et al. [18], the survival rate in ALF pigs was significantly improved by this treatment. Furthermore, several studies indicated that the portal vein transplantation route exhibited distinct advantages. Cao et al. [19] and Cen et al. [20] demonstrated that this route significantly prolonged the survival time in porcine models of ALF. Sang et al. [21] further confirmed its superiority over the peripheral vein transplantation route in improving liver function parameters. Wang et al. [8] systematically summarized the progress in mesenchymal stem cell-based therapy for the treatment of acute liver failure, agreeing that MSCs have therapeutic potential in improving the survival rate and liver function in pigs with ALF. Although MSC therapy is effective in large animals, the sample sizes have been limited due to the experimental costs and other factors, affecting the reliability of translating these findings to clinical settings.

Therefore, we performed a meta-analysis to provide a more comprehensive and quantitative understanding of the therapeutic effects and safety of MSC therapy in large animals with ALF to aid in clinical practice.

## 2. Results

### 2.1. Study Inclusion

**Figure 1:**
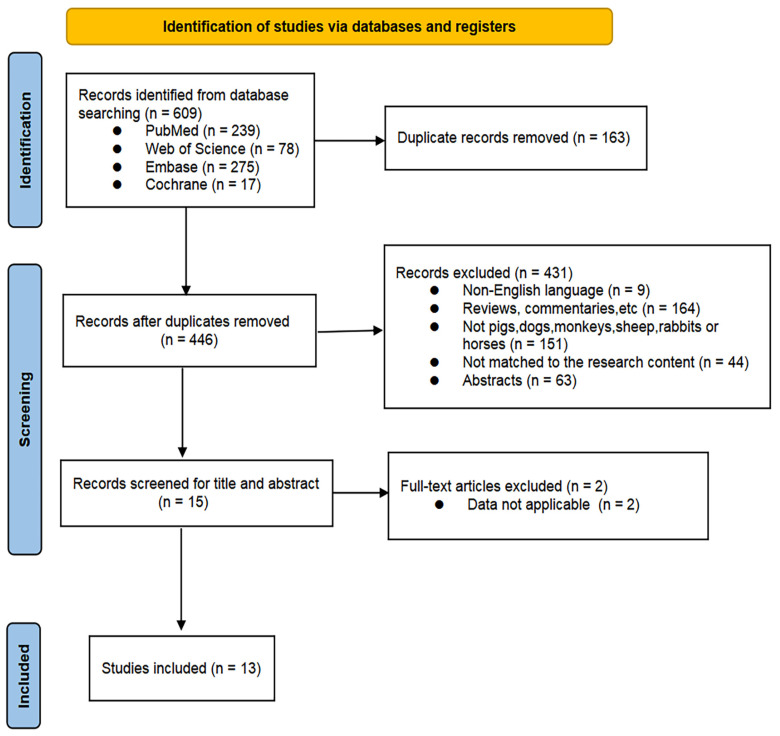
Literature screening flowchart.

In total, 609 potentially relevant articles on MSC therapy for large animal models with ALF were identified from the databases, including 239 from PubMed/Medline, 78 from Web of Science, 275 from Embase, and 17 from Cochrane Library. After removing duplicates, 446 records were retained. Following the review of titles and abstracts, fifteen records were retained. Finally, after reviewing the full texts, the references, and the cited literature, 13 studies were included. The process for the literature screening is presented in Figure 1.

### 2.2. Included Study Characteristics

The characteristics of the 13 studies are presented in Table 1. Large animal models that included pigs (n = 11) and monkeys (n = 2) were employed in these investigations published between 2012 and 2024. The modeling methods included D-galactosamine (D-gal) injection (n = 10), α-amatoxin and lipopolysaccharide (LPS) injection (n = 1), α-amanitin injection (n = 1), and I-R injury (n = 1). MSCs were sourced from hBMSCs (n = 4), pBM-MSCs (n = 3), human umbilical cord mesenchymal stem cells (hUC-MSCs) (n = 2), MenSCs (n = 1), hPMSCs (n = 2), and human adipose-derived MSCs (n = 1). MSCs were administered via deep vein injection routes, including portal vein and splenic vein injection (n = 8), as well as via peripheral vein transplantation routes, including the jugular vein, ear vein, external ear vein, and peripheral infusion (n = 2). Both transplantation methods were also mentioned in MSC transplantation (n = 3). The MSC doses ranged from 1 × 10^7^ to 1 × 10^8^, with single (n = 12) and multiple (n = 1) injections. This heterogeneity led to the fragmentation of the evidence base. Consequently, the subsequent analyses in this study regarding the impact of specific variables such as cell source, dosage, and route of administration on therapeutic efficacy are exploratory in nature.

### 2.3. Quality Assessment

**Figure 2:**
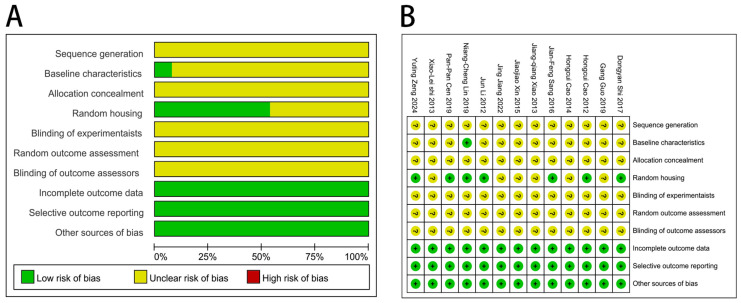
Risk of bias assessment: (**A**) Risk of bias among the studies considered. Different colors (green, red, yellow) represent “low risk of bias”, “high risk of bias”, and “unclear risk of bias”, respectively. (**B**) Risk of bias summary among the studies considered. Different symbols (“+”, “?”) denote “low risk of bias” and “unclear risk of bias”, respectively [16,17,18,19,20,21,22,23,24,25,26,27,28].

Figure 2 shows the quality evaluation of the 13 included studies. Ten studies mentioned randomization, while three did not mention it. None of the studies reported the specific method. One study presented identical baseline characteristics, while the remaining twelve provided insufficient descriptions of the baseline characteristics across groups, making it impossible to determine whether they were identical. Seven studies mentioned that animals were randomly housed, but none of the thirteen studies mentioned allocation concealment, blinding of relevant personnel, or whether the animals were randomly selected when evaluating the results. All studies reported complete data, which was considered unrelated to selective reporting, and no additional sources of bias were identified. In summary, these studies did not present a high risk of bias.

### 2.4. Meta-Analysis of Outcomes

Thirteen articles were included for meta-analysis, with the survival rate, ALT, AST, IL-6, and TNF-α as the indicators to assess the efficacy of MSC for large animals with ALF.

#### 2.4.1. Survival Rate

**Figure 3:**
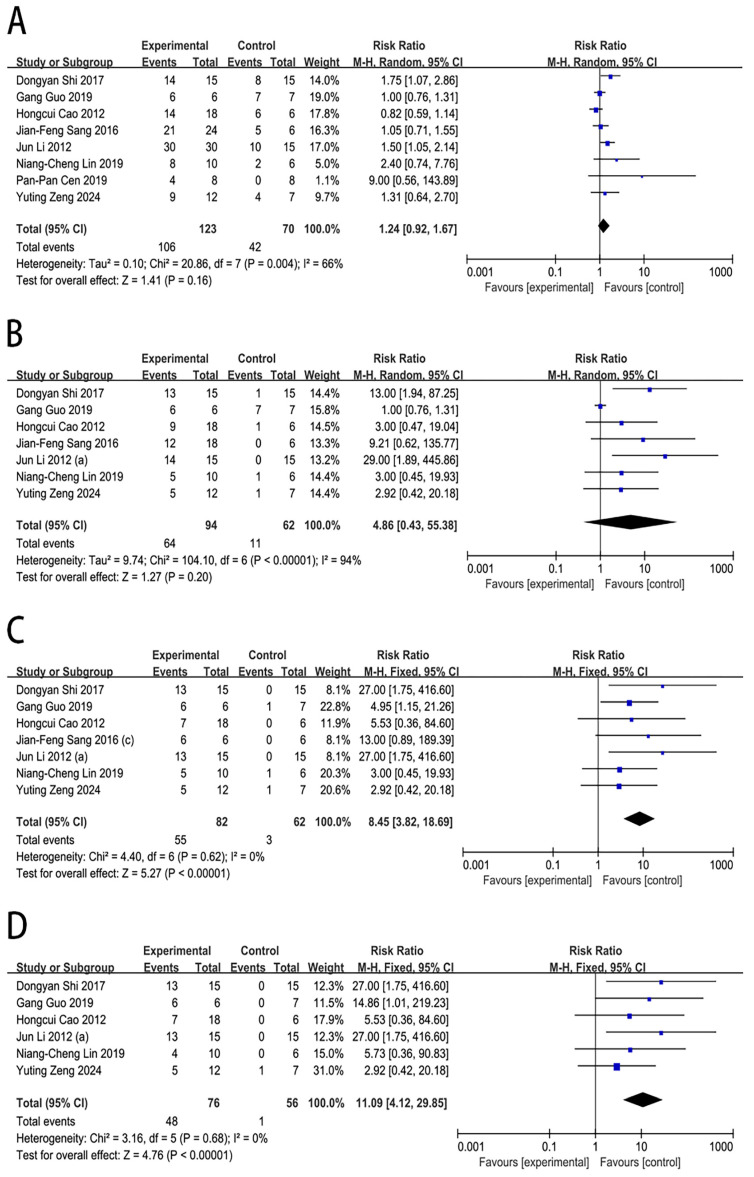
Forest plot of the effect of MSC therapy on the survival rate: (**A**) Survival rate at 3 days. (**B**) Survival rate at 5 days. (**C**) Survival rate at 7 days. (**D**) Survival rate at 14 days [16,17,18,19,20,21,22,27].

The effect of MSC therapy on the survival rate of large animals with ALF was reported in eight studies. In comparison to the control group, MSC transplantation was associated with a significantly increased survival rate at 7 days (RR = 8.45, 95% CI [3.82, 18.69], *p* < 0.00001) and 14 days (RR = 11.09, 95% CI [4.12, 29.85], *p* < 0.00001). However, no significant differences were observed at 3 days (RR = 1.24, 95% CI [0.92, 1.67], *p* = 0.16) and 5 days (RR = 4.86, 95% CI [0.43, 55.38], *p* = 0.20), as shown in Figure 3.

Substantial heterogeneity was observed at 3 days (I^2^ = 66%) and 5 days (I^2^ = 94%). In the sensitivity analysis for the survival rate in the 5-day subgroup (Supplementary File S2: Table S2), the exclusion of the study by Guo et al. [16] resulted in a marked reduction in heterogeneity. The I^2^ statistic decreased to 0% among the remaining studies, suggesting that this study may be the main driver of the heterogeneity observed in these subgroups. There was no significant publication bias for the survival rate at 3 days (Egger’s test: *p* = 0.062) and 7 days (Egger’s test: *p* =0.086). However, publication bias was observed for the survival rate at 5 days (Egger’s test: *p* = 0.002), as assessed by the funnel plot and Egger’s test (Supplementary File S3).

#### 2.4.2. ALT Level

**Figure 4:**
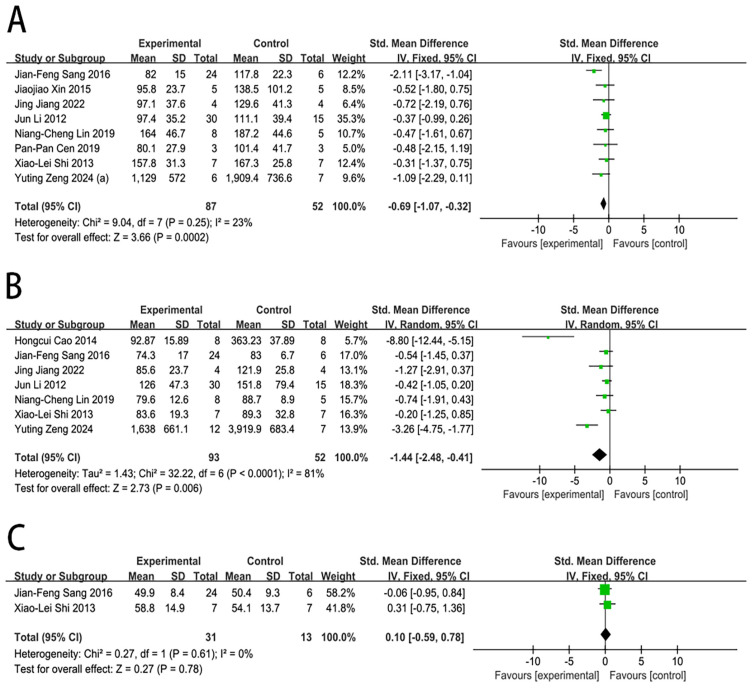
Forest plot of the effect of MSC therapy on the ALT level: (**A**) ALT level at 1 day. (**B**) ALT level at 3 days. (**C**) ALT level at 5 days [17,20,21,22,24,25,26,27,28].

Nine studies reported the impact of MSC transplantation on the ALT level in large animals with ALF. In contrast with the control group, MSC transplantation was associated with a significantly lower ALT level at 1 day (SMD = −0.69, 95% CI [−1.07, −0.32], *p* = 0.0002) and 3 days (SMD = −1.44, 95% CI [−2.48, −0.41], *p* = 0.006). However, the ALT level at 5 days (SMD = 0.10, 95% CI [−0.59, 0.78], *p* = 0.78) did not differ significantly from those in the control group, as shown in Figure 4.

Substantial heterogeneity was observed at 3 days (I^2^ = 81%). In the sensitivity analysis for the ALT level in the 3-day subgroup (Supplementary File S2: Table S4), the exclusion of Cao et al. [25]’s study resulted in a marked reduction in heterogeneity. The I^2^ statistic decreased to 63% among the remaining studies, suggesting that this study may be the main driver of the heterogeneity observed in this subgroup. There was no significant publication bias for the ALT level at 1 day (Egger’s test: *p* = 0.548). However, publication bias was observed for the ALT level at 3 days (Egger’s test: *p* = 0.021), as assessed by the funnel plot and Egger’s test (Supplementary File S3).

#### 2.4.3. AST Level

**Figure 5:**
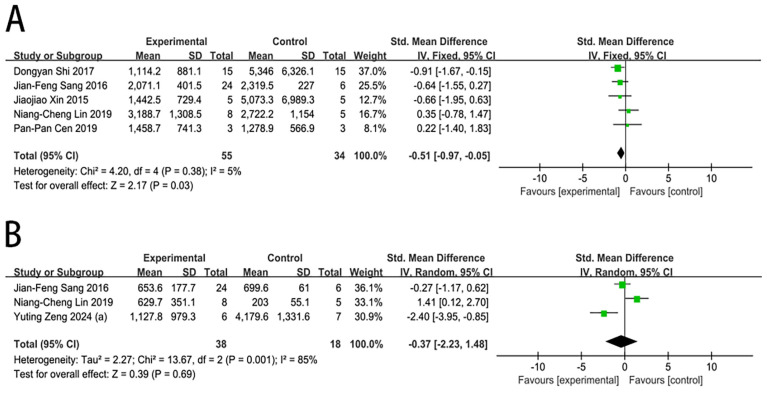
Forest plot of the effect of MSC therapy on the AST level: (**A**) AST level at 1 day. (**B**) AST level at 3 days [17,18,20,21,26,27].

Six studies reported the impact of MSC transplantation on the AST level in large animals with ALF. In contrast to the control group, MSC transplantation was associated with a significantly lower AST level at 1 day (SMD = −0.51, 95% CI [−0.97, −0.05], *p* = 0.03). However, no significant differences were found at 3 days (SMD = −0.37, 95% CI [−2.23, 1.48], *p* = 0.69), as shown in Figure 5.

Substantial heterogeneity was observed at 3 days (I^2^ = 85%). In the sensitivity analysis for the AST level in the 3-day subgroup (Supplementary File S2: Table S6), with no significant reduction in heterogeneity within this subgroup after sequentially removing individual studies. Due to the limited number of studies on AST levels, publication bias was not assessed.

#### 2.4.4. IL-6 and TNF-α Levels

Due to the limited number of studies available, a meta-analysis for IL-6 and TNF-α levels was not performed, and the findings are summarized descriptively. Regarding IL-6, data from two studies at different time points were reported. Zeng et al. [17] indicated that IL-6 level in a rhesus monkey model began to increase at 12–24 h after liver injury. Xiao et al. [23] reported that IL-6 level in the MSC transplantation group increased rapidly within 24 h after D-gal induction and remained elevated for several days before declining slowly. Compared with the control group, IL-6 level in the MSC group was lower at later time points; however, the differences were not statistically significant. For TNF-α, data were available from one study by Xiao et al. [23]. The overall trend and between-group comparisons were similar to those reported for IL-6 in the same study.

### 2.5. Subgroup Analysis

We performed subgroup analyses to investigate whether the effects of MSC therapy on the survival rate and ALT and AST levels at various time points were influenced by the animal model, source of MSCs, delivery route, and dose. Due to insufficient eligible studies, subgroup analyses of the IL-6 and TNF-α levels were not conducted.

#### 2.5.1. Analysis of Animal Models

In pig models, the survival rate was significantly improved at 5 days (RR = 8.53, 95% CI [3.42, 21.27], *p* < 0.00001), 7 days (RR = 11.86, 95% CI [4.09, 34.43], *p* < 0.00001), and 14 days (RR = 14.75, 95% CI [3.97, 54.81], *p* < 0.0001), whereas in monkey models, the survival rate was significantly improved at 7 days (RR = 3.99, 95% CI [1.25, 12.71], *p* = 0.02) and 14 days (RR = 6.14, 95% CI [1.38, 27.27], *p* = 0.02) (Supplementary File S2: Table S1). In pig models, ALT levels were significantly lower than those in the control group at 1 day (SMD = −0.65, 95% CI [−1.04, −0.26], *p* = 0.001) and 3 days (SMD = −1.04, 95% CI [−2.01, −0.08], *p* = 0.03) post-transplantation. Similarly, AST levels were significantly reduced at 1 day (SMD = −0.51, 95% CI [−0.97, −0.05], *p* = 0.03) (Supplementary File S2: Tables S3 and S5).

#### 2.5.2. Pig Model Subgroup Analysis

Based on the tissue sources of MSCs, subgroup analysis was performed. The survival rate was significantly improved in the BM-MSC group at 3 days (RR = 1.45, 95% CI [1.14, 1.84], *p* = 0.003), 5 days (RR = 15.34, 95% CI [4.02, 58.53], *p* < 0.0001), 7 days (RR = 22.33, 95% CI [4.63, 107.72], *p* = 0.0001), and 14 days (RR = 27.00, 95% CI [3.90, 186.92], *p* = 0.0008). However, no significant difference in survival rate was found in the non-BM-MSC group (Supplementary File S2: Table S1). The ALT level was significantly reduced in the BM-MSC group at 1 day (SMD = −0.78, 95% CI [−1.46, −0.10], *p* = 0.02) and 3 days (SMD = −0.47, 95% CI [−0.92, −0.03], *p* = 0.04), but no significant difference was found for the non-BM-MSC group (Supplementary File S2: Table S3). In contrast to the control group, the BM-MSC group exhibited a significant reduction in the AST level at 1 day (SMD = −0.78, 95% CI [−1.31, −0.25], *p* = 0.004) in pig models of ALF, but the non-BM-MSC group did not show any significant difference. The heterogeneity of AST levels in the BM-MSC group and the non-BM-MSC group was substantially reduced, with I^2^ decreasing to 0% (Supplementary File S2: Table S5).

In the subgroup analysis based on different transplantation routes, the survival rate was significantly improved in the deep vein transplantation group at 5 days (RR = 9.40, 95% CI [3.77, 23.47], *p* < 0.00001), 7 days (RR = 12.29, 95% CI [4.21, 35.90], *p* < 0.00001), and 14 days (RR = 15.50, 95% CI [4.12, 58.39], *p* < 0.0001). However, no significant difference in survival rate was found in the peripheral vein transplantation group (Supplementary File S2: Table S1). Compared with the control group, the deep vein transplantation group exhibited a significant reduction in the ALT level at 1 day (SMD = −0.67, 95% CI [−1.11, −0.24], *p* = 0.002) and 3 days (SMD = −1.70, 95% CI [−2.95, −0.46], *p* = 0.007), but no significant difference was found for the peripheral vein transplantation route (Supplementary File S2: Table S3). In comparison with the control group, the deep vein transplantation group showed a significant reduction in the AST level at 1 day (SMD = −0.58, 95% CI [−1.08, −0.09], *p* = 0.02). Analysis was not possible for the peripheral vein transplantation group due to an insufficient number of studies (Supplementary File S2: Table S5).

Subgroup analysis based on different doses revealed the following results. Animals that received a dose of (0.7–2.5) × 10^6^/kg showed a significant improvement in the survival rate at 5 days (RR = 5.29, 95% CI [1.08, 25.92], *p* = 0.04) and 7 days (RR = 5.86, 95% CI [1.31, 26.14], *p* = 0.02), while the (3.0–3.3) × 10^6^/kg group showed this benefit at 3 days (RR = 1.59, 95% CI [1.19, 2.13], *p* = 0.002), 5 days (RR = 18.33, 95% CI [3.83, 87.84], *p* = 0.0003), 7 days (RR = 27.00, 95% CI [3.90, 186.92], *p* = 0.0008), and 14 days (RR = 27.00, 95% CI [3.90, 186.92], *p* = 0.0008) compared to their respective control groups. Analysis was not possible for the (6.7–10) × 10^6^/kg group due to an insufficient number of studies (Supplementary File S2: Table S1). Neither the (0.7–2.5) × 10^6^/kg group, nor the (3.0–3.3) × 10^6^/kg group, nor the (6.7–10) × 10^6^/kg group showed a significant reduction in the ALT level compared to the control group (Supplementary File S2: Table S3). The (3.0–3.3) × 10^6^/kg group exhibited a significant reduction in the AST level on day 1 (SMD = −0.85, 95% CI [−1.50, −0.19], *p* = 0.01) post-transplantation in comparison with the control group. The heterogeneity was substantially reduced in the (3.0–3.3) × 10^6^/kg group, with I^2^ decreasing to 0%. Compared with the control group, no significant difference was found in the (0.7–2.5) × 10^6^/kg group. Analysis was not possible for the (6.7–10) × 10^6^/kg group due to an insufficient number of studies (Supplementary File S2: Table S5).

### 2.6. Sensitivity Analysis

To verify the robustness of the analytical findings, we conducted a set of sensitivity analyses. As all modeling methods other than D-gal involved only a single study each, we performed a sensitivity analysis after excluding them (Supplementary File S2: Table S7). The results demonstrated that improvements in the survival rates reached statistical significance at 5 days, 7 days, and 14 days, with the effect size increasing over time. Regarding liver function, the ALT level was reduced in the early phase, but this effect waned by day 5. Changes in the AST were consistent in direction with the primary analysis, although the magnitude of the effect was attenuated. In summary, sensitivity analyses indicate that the efficacy trend of MSC therapy in D-gal-induced large animal models of ALF remains broadly consistent with the primary analysis. However, the data for certain time points are limited or sensitive to individual studies, indicating that the current conclusions remain exploratory and require validation by further high-quality studies.

### 2.7. Adverse Events and Safety Assessment

Current preclinical research data indicate that MSC transplantation exhibits excellent safety characteristics in large animal models. Li et al. [22] first demonstrated that hBMSC portal vein transplantation is a safe and efficient treatment method in a large animal model of pigs with fulminant hepatic failure (FHF). According to a comprehensive analysis of the literature, infusion via either the portal or peripheral vein has not been associated with reports of acute complications, such as thromboembolism. However, Cao et al. [19] reported that hPMSC peripheral vein infusion was associated with fever and adverse immune reactions.

Long-term follow-up studies also provide critical evidence for safety assessment. Zeng et al. [17] conducted a five-year follow-up study on rhesus monkeys, confirming that no cases of carcinogenesis, organ fibrosis, thrombosis, or immune system dysfunction were observed in monkeys treated with allogeneic hUC-MSCs, with most serum markers maintained within normal reference ranges. This conclusion aligns with the findings of Guo et al. [16], who reported no tumors in rhesus monkeys treated with hUC-MSCs over a three-year follow-up period. Furthermore, the observed tolerance of rhesus monkeys to allogeneic human cells provides additional confirmation of the low immunogenicity of hUC-MSCs.

## 3. Discussion

There has been significant progress in the study of using MSCs for liver failure, including ALF and acute on chronic liver failure (ACLF). The effectiveness of MSC treatment for liver failure is supported by evidence from both animal studies and clinical trials. These results include significant improvements in liver function indicators and increased patient survival rates [29,30,31]. Several previous meta-analysis studies also demonstrated that MSC therapy is an effective and safe treatment approach for patients with ACLF, with the most common adverse reaction being fever only and no severe side effects reported [32,33]. MSCs are adult stem cells derived from the mesoderm. They have high self-renewal capacity and are therefore considered the most promising cells for liver failure treatment. The main objective of our study was to assess the therapeutic effects and safety of MSC therapy in large animals with ALF. The results of this meta-analysis indicated that MSC treatment can alleviate the levels of ALT and AST. In large animals with ALF, MSC therapy was associated with a significantly improved survival rate. There were no serious adverse events after implantation of stem cells, with the most common adverse reaction being fever only. Overall, this study indicates that MSC therapy is a safe and potential therapeutic option for large animals with ALF.

The various repair mechanisms of MSCs may explain their therapeutic efficacy. They modulate inflammatory factors through paracrine actions such as upregulating IL-10 and downregulating TNF-α and IL-6 [34,35,36]. Meanwhile, they possess strong regulatory capabilities over immune cells, including macrophages and T cells [37]. MSCs can migrate to injured livers through their homing ability [38] and reduce cytotoxic responses and ROS generation through their antioxidant properties, effectively decreasing the serum ALT and AST levels and protecting the liver [39].

It has also been reported that the liver disease type, cell source, injection route, and injection times can apparently influence the efficacy of MSC therapy in different chronic liver diseases [40]. To further explore the factors influencing the efficacy of MSC therapy for ALF, we performed subgroup analyses. First, the subgroup analysis was stratified by animal species. Compared with the control group, ALT levels in pig models were significantly decreased on days 1 and 3, while AST levels showed a significant decrease only on day 1. MSC therapy significantly improved survival in pig models on days 5, 7, and 14. In monkey models, a significant improvement in survival was observed at 7 days and 14 days. This divergence may be attributed to inherent physiological and metabolic differences between pigs and non-human primates. Due to their size and physiological similarity to humans, pigs may exhibit a more rapid response to MSC infusion. Owing to the limited number of studies on non-human primate models, the following in-depth discussion on the MSC source, transplantation route, and cell dosage is based on data from pig models.

The source of MSCs significantly impacts their therapeutic efficacy [41]. One subgroup analysis compared BM-MSCs with non-BM-MSCs, including MenSCs, hPMSCs, and human adipose-derived MSCs. BM-MSCs have been the focus of extensive research; however, the other three sources offer advantages such as relatively non-invasive or minimally invasive collection and greater convenience [42,43,44]. Compared with the control group, the BM-MSC group showed a significant improvement in survival rate at all time points. AST and ALT levels were significantly reduced on day 1, with the protective effect most evident during the early treatment phase. But neither the reduction in ALT and AST nor the improvement in survival rate by non-BM-MSCs reached statistical significance. Therefore, in clinical scenarios where both survival rate improvement and liver protection are desired, BM-MSCs may be a more suitable candidate.

The included studies primarily involved transplanting MSCs into the body through peripheral or deep veins, with peripheral vein injection a common method [45]. Compared with deep vein transplantation routes such as portal vein transplantation, peripheral vein transplantation routes offer several advantages, including operational simplicity and low risk. In this study, the deep vein transplantation route significantly improved the survival rate at most time points in pig models with ALF, while the peripheral vein transplantation route showed no statistically significant improvement. Regarding the improvement in liver injury markers, the deep vein transplantation route showed a rapid reduction in ALT and AST levels in the early phase, while the peripheral vein transplantation route showed no significant reduction in ALT, and data for AST levels were not sufficient. These findings suggest that the deep vein transplantation route may be a more promising strategy, with the difference in efficacy likely due to differences in the MSC delivery efficiency between the two routes. The deep vein transplantation route achieved efficient homing of MSCs to the liver, allowing the majority of MSCs to reach the hepatic tissue. In contrast, peripheral vein transplantation resulted in significant pulmonary sequestration, with only a minimal fraction of cells reaching the liver [46]. However, the deep vein transplantation route carries risks due to its invasive nature. Further clinical trials are needed to compare the treatment outcomes of transplantation route choices while also considering the patient’s condition and status and balancing the risk–benefit ratio.

The MSC dosage is a core issue in cell transplantation. It directly impacts treatment safety and efficacy and the establishment of the optimal treatment regimen. This study found that MSC doses of (0.7–2.5) × 10^6^/kg and (3.0–3.3) × 10^6^/kg significantly improved the survival rate in large animal models with ALF, whereas data for the 6.7–10 × 10^6^/kg dose were insufficient. Although the (0.7–2.5) × 10^6^/kg, (3.0–3.3) × 10^6^/kg, and (6.7–10) × 10^6^/kg dose groups showed no significant reduction in ALT levels, the (3.0–3.3) × 10^6^/kg group showed high homogeneity (I^2^ = 0%), indicating a consistent trend across studies. Only a dose of (3.0–3.3) × 10^6^/kg showed a significant reduction in the AST level, while the (0.7–2.5) × 10^6^/kg dose showed no notable difference compared with the control group. Therefore, we hypothesize that while a specific range of MSC doses can significantly ameliorate liver injury, beyond this range, the therapeutic benefit ceases to be significant, and the outcome uncertainty rises. A similar phenomenon was reported by Liu et al. [47], who observed that the therapeutic effect of MSCs on the survival rate reached a plateau beyond a certain cell dose in a mouse model of acute liver failure. Furthermore, administration of high doses may also carry certain risks, including an increased risk of thrombosis [48]. Analysis of the survival rate and liver injury markers suggests that a dose range of (3.0–3.3) × 10^6^/kg may be a potentially effective dosage. However, given the limited number of included studies, future research requires more independent large-scale studies to validate the correlation between the transplant dose and therapeutic efficacy.

This study had some limitations. (1) The primary limitation of the study lies in the methodological quality of the included studies. None of the 13 studies reported allocation concealment, blinding procedures, or specific randomization methods. Ten studies mentioned randomization but provided no details. Publication bias was detected for several outcomes, which may have influenced the results. Therefore, the findings of this study require further confirmation by higher-quality literature. (2) Some of the outcome measures exhibited high heterogeneity. Although subgroup analyses were conducted, some outcome measures still exhibited significant heterogeneity, which should be considered seriously. Due to this heterogeneity, the statistical results of this study may deviate from the actual values. (3) The limited sample size of large animal models in these studies may have impacted the results of this research.

This meta-analysis also has distinct advantages. To the best of our knowledge, this study provides the first comprehensive meta-analysis of the role of MSC therapy in large animal models with ALF. This study provides preliminary evidence that may serve as a useful reference for considering critical variables, such as MSC source and dosage, when designing clinical trials for ALF.

## 4. Materials and Methods

This meta-analysis was carefully designed and performed in accordance with the PRISMA 2020 guidelines [49]. The protocol of this study was prospectively registered on the PROSPERO website with registration number CRD420251002844 (http://www.crd.york.ac.uk/prospero/display_record.php?ID=CRD420251002844), accessed on 4 March 2025.

### 4.1. Search Strategy

A literature search was conducted in Web of Science, Embase, Cochrane Library, and PubMed/Medline for eligible articles published up to 3 March 2025. Subject terms such as “Mesenchymal stem cells” and “Acute liver failure” were used to identify diseases and interventions. The database search method is detailed in Supplementary File S1.

### 4.2. Inclusion and Exclusion Criteria

The original literature used for this meta-analysis met the subsequent screening requirements: (1) large animal models of ALF, including pigs, monkeys, dogs, sheep, rabbits, or horses, with no gender restrictions; (2) treatment involving MSCs at any dose, timing, or frequency; (3) control groups using ALF models, treated with distilled water, saline, or the same solvent, or those receiving no treatment; (4) included outcome measures: survival rate, ALT, AST, IL-6, and TNF-α.

The exclusion criteria were any of the following: (1) non-original research papers, such as reviews, conference abstracts, or editorials; (2) research not published in English; (3) animal models not including dogs, pigs, monkeys, sheep, rabbits, or horses; animal models without ALF; non-animal experiments; (4) studies with no control group, case studies, or crossover studies; (5) large animal models of ALF treated with substances other than MSCs; (6) control groups treated with substances other than purified water, saline, or the same solvent and control groups without an ALF model; (7) full text unavailable or studies with insufficient data.

### 4.3. Data Extraction

Two reviewers (Y.Z. and Y.Y.) performed the literature screening and data extraction independently. EndNote 21 was used to remove duplicate entries from the retrieved literature. Titles and abstracts were screened according to the eligibility criteria to exclude irrelevant studies. The remaining articles underwent full-text review to assess their eligibility for inclusion. Numerical data were first extracted from the tables or text during data extraction. If these data were not reported, data were extracted from charts using WebPlotDigitizer [50]. If there was uncertainty or missing data in the article, the authors of the article were contacted via email.

The extracted information was as follows: (1) first author and publication year; (2) species, gender, and weight of the experimental animals and methods for creating ALF animal models; (3) number of animals in the experimental and control groups; (4) source of MSCs, transplantation route, and timing and dose of MSC transplantation after modeling; (5) primary outcome measures: survival rate, ALT, and AST; secondary outcome measures: IL-6 and TNF-α.

We also constructed descriptive summary tables for the key methodological characteristics and results of each extracted study. The structured summaries of these extracted data were summarized (Supplementary File S4: Tables S1–S5). The aim is to provide readers with the necessary background information for interpreting the pooled results of this meta-analysis and to clearly demonstrate the distribution and sources of heterogeneity in the existing evidence for each study.

### 4.4. Quality Assessment

Review Manager 5.3 was used for quality assessment. Each included study was independently assessed by two reviewers (Y.Z. and Y.Y.) using the SYRCLE animal study risk of bias assessment tool. Any disagreements were settled by consensus. The evaluation outcomes comprised low, uncertain, and high bias risk.

### 4.5. Statistical Analysis

Review Manager 5.3 and STATA 15 were employed to conduct the statistical analysis. Continuous data are presented as standardized mean differences (SMDs) and 95% confidence intervals (CIs). For dichotomous survival data, with survival defined as the event, the results are presented as risk ratios (RRs) and 95% CIs. A *p*-value below 0.05 was considered statistically significant. I2 was employed to evaluate the heterogeneity, and a random-effects model was used if I^2^ > 50%, indicating significant statistical heterogeneity. Otherwise, a fixed-effects model was employed. In this study, some trials with multiple intervention arms and a shared control group were included. To avoid double-counting control data, the intervention arms were pooled. Analyses of subgroups and sensitivity were conducted to examine potential sources of heterogeneity. Due to distinct physiological structures and immune responses between pigs and monkeys, we conducted subgroup analyses based on animal models. For the pig models (11 studies), we investigated the effects of the MSC source, dose, and transplantation route on MSC efficacy. In contrast, only two studies included monkey models, and subgroup analysis was not performed due to insufficient data. To examine the impact of the MSC source on the therapeutic outcomes, and considering the limited number of studies for certain sources, we categorized cell sources into two subgroups: (1) the BM-MSC group, including human bone marrow mesenchymal stem cells (hBMSCs) and porcine bone marrow mesenchymal stem cells (pBM-MSCs); and (2) the non-bone marrow mesenchymal stem cell (non-BM-MSC) group, including menstrual blood stem cells (MenSCs), human placental mesenchymal stem cells (hPMSCs), and human adipose mesenchymal stem cells. To evaluate the effect of the cell dosage, we divided the studies into three subgroups based on the distribution of dosages in the included studies: (0.7–2.5) × 10^6^/kg, (3.0–3.3) × 10^6^/kg, and (6.7–10) × 10^6^/kg. For studies reporting the total dosage, we uniformly converted it into the dosage per kilogram of body weight. If the animal body weight was given as a range, we used the mean value for conversion.

## 5. Conclusions

The findings show that MSC therapy can improve survival rate and liver function and alleviate clinical symptoms without serious adverse events in large animal models of ALF. However, these conclusions are drawn from studies with methodological limitations and unclear risk of bias, and the findings need to be confirmed in future studies. Key questions remain, including the optimization of the cell source, cell dosage, injection frequency, transplantation route, and the type of large animal model, which must be addressed before routine clinical application. Further evaluation and validation of the safety and efficacy of MSCs for ALF in clinical applications are still needed through large-scale and high-quality clinical RCTs.

## Figures and Tables

**Table 1:** Basic characteristics of the included studies.

First Author	Species	Sex	Weight (kg)	No. of Treated Animals	No. of Control	Model Method	Source of MSCs	Control Group	Delivery Route	Dose	Time of Injection (h)	Outcomes
Hongcui Cao 2012 [19]	Pig	Male	10–12	18	6	1.5 g/kg D-Gal	hPMSCs; X-ray-treated hPMSCs	Not reported	Jugular venous catheter or the portal vein	1 × 10^8^	18	Survival rate
Jun Li 2012 [22]	Pig	Male	8–10	30	15	1.5 g/kg D-Gal	hBMSCs	Saline	Intrahepatic portal vein; ear vein	3 × 10^7^	Not reported	Survival rate; ALT
Jiang-qiang Xiao 2013 [23]	Swine	Not reported	7–13	7	7	0.3 g/kg D-Gal	pBM-MSCs	Saline	Portal vein	8 × 10^7^	24	IL-6; TNF-α
Xiao-Lei Shi 2013 [24]	Swine	Not reported	7–13	7	7	0.3 g/kg D-Gal	pBM-MSCs	Saline	Portal vein	1 × 10^8^	24	ALT
Hongcui Cao 2014 [25]	Pig	Male	10–12	8	8	1.5 g/kg D-Gal	hPMSCs	Saline	Intrahepatic portal vein	1 × 10^8^	18	ALT
Jiaojiao Xin 2015 [26]	Pig	Not reported	8–10	5	5	1.5 g/kg D-Gal	hBMSCs	Saline	Intrahepatic portal vein	3 × 10^7^	Immediate	ALT; AST
Jian-Feng Sang 2016 [21]	Swine	Not reported	12–18	24	6	0.3 g/kg D-Gal	pBM-MSCs	Saline	External ear vein; portal vein; arteria hepatica or intrahepatic	1 × 10^7^	24	Survival rate; ALT; AST
Dongyan Shi 2017 [18]	Pig	Male	8–10	15	15	1.5 g/kg D-Gal	hBMSCs	Saline	Intrahepatic portal vein	3 × 10^6^ /kg	Immediate	Survival rate; AST
Pan-Pan Cen 2019 [20]	Pig	Male	8–12	20	20	1.0 g/kg D-Gal	MenSCs	Saline	Portal vein	2.5 × 10^6^ /kg	Immediate	Survival rate; ALT; AST
Niang-Cheng Lin 2019 [27]	Pig	Male	10–15	10	6	I-R Injury	Human adipose-derived MSCs	Saline	Splenic vein	2.4 × 10^7^	Not reported	Survival rate; ALT; AST
Gang Guo 2019 [16]	Monkey	Male/ Female	5.1–7.2	6	7	α-amatoxin (25 μg/kg) and LPS (1 μg/kg)	hUC-MSCs	Saline	Peripheral infusion	1 × 10^7^	2, 24, 48	Survival rate
Jing Jiang 2022 [28]	Pig	Male	8–10	5	5	1.5 g/kg D-Gal	hBMSCs	Saline	Intrahepatic portal vein	3 × 10^6^ /kg	Not reported	ALT
Yuting Zeng 2024 [17]	Monkey	Male	5.5–8.3	12	7	40 μg/kg α-amanitin	hUC-MSCs	Saline	Peripheral infusion	1 × 10^7^; 2 × 10^7^	48	Survival rate; ALT; AST; IL-6

## Data Availability

The original contributions presented in this study are included in the article/Supplementary Materials. Further inquiries can be directed to the corresponding author.

## Supplementary Materials

The following supporting information can be downloaded at: https://www.mdpi.com/article/10.3390/ijms27073175/s1.

## Abbreviations

The following abbreviations are used in this manuscript:

MSCs	mesenchymal stem cells
ALF	acute liver failure
ALT	alanine aminotransferase
AST	aspartate aminotransferase
BM-MSCs	bone marrow-derived mesenchymal stem cells
RCTs	randomized controlled trials
OLT	orthotopic liver transplantation
SMDs	standardized mean differences
CIs	confidence intervals
RRs	risk ratios
hBMSCs	human bone marrow mesenchymal stem cells
pBM-MSCs	porcine bone marrow mesenchymal stem cells
non-BM-MSC	non-bone marrow mesenchymal stem cell
MenSCs	menstrual blood stem cells
hPMSCs	human placental mesenchymal stem cells
D-gal	D-galactosamine
LPS	lipopolysaccharide
hUC-MSCs	human umbilical cord mesenchymal stem cells
