# Supplementary material for: The Efficacy of Mesenchymal Stem Cell Therapy in Large Animal Models of Acute Liver Failure: A Meta-Analysis
Source: Int J Mol Sci. 2026 Mar 31;27(7):3175. doi: 10.3390/ijms27073175 (PMC13072887; doi:10.3390/ijms27073175)
Supplement: Supplementary file 1 [file ijms-27-03175-s001.zip › Supplementary File S1. Search strategies.pdf]

Supplementary File S1. Search strategies

	Search Method (PubMed)
#1	"Mesenchymal Stem Cells"[Mesh]
#2	((Stem Cell, Mesenchymal) OR (Mesenchymal Stem Cell)) OR (Stem Cells, Mesenchymal)) OR (Mesenchymal Stromal Cells)) OR (Mesenchymal Stromal Cell)) OR (Stromal Cell, Mesenchymal)) OR (Stromal Cells, Mesenchymal)) OR (Wharton Jelly Cells)) OR (Wharton's Jelly Cells)) OR (Wharton's Jelly Cell)) OR (Whartons Jelly Cells)) OR (Bone Marrow Stromal Cells)) OR (Bone Marrow Stromal Cell)) OR (Bone Marrow Stromal Cells, Multipotent)) OR (Multipotent Bone Marrow Stromal Cell)) OR (Multipotent Bone Marrow Stromal Cells)) OR (Bone Marrow Stromal Stem Cells)) OR (Mesenchymal Progenitor Cell)) OR (Mesenchymal Progenitor Cells)) OR (Progenitor Cell, Mesenchymal)) OR (Progenitor Cells, Mesenchymal)) OR (Multipotent Mesenchymal Stromal Cells)) OR (Mesenchymal Stromal Cells, Multipotent)) OR (Multipotent Mesenchymal Stromal Cell)) OR (Bone Marrow Mesenchymal Stem Cells)) OR (Bone Marrow Mesenchymal Stem Cell)) OR (Adipose-Derived Mesenchymal Stem Cells)) OR (Adipose Derived Mesenchymal Stem Cells)) OR (Adipose-Derived Mesenchymal Stromal Cells)) OR (Adipose Derived Mesenchymal Stromal Cells)) OR (Mesenchymal Stem Cells, Adipose-Derived)) OR (Mesenchymal Stem Cells, Adipose Derived)) OR (Adipose Tissue-Derived Mesenchymal Stromal Cell)) OR (Adipose Tissue Derived Mesenchymal Stromal Cell)) OR (Adipose Tissue-Derived Mesenchymal Stromal Cells)) OR (Adipose Tissue Derived Mesenchymal Stromal Cells)) OR (Adipose Tissue-Derived Mesenchymal Stem Cell)) OR (Adipose Tissue Derived Mesenchymal Stem Cell)) OR (Adipose Tissue-Derived Mesenchymal Stem Cells)) OR (Adipose Tissue Derived Mesenchymal Stem Cells)) OR (Adipose-Derived Mesenchymal Stem Cell)) OR (Adipose Derived Mesenchymal Stem Cell)) OR (Umbilical Cord-derived Mesenchymal Stem Cells)) OR (Human Mesenchymal Stem Cells)) OR (Placenta-derived Mesenchymal Stem Cells)
#3	#1 OR #2
#4	"Liver Failure, Acute"[Mesh]
#5	((((((((((((((((Failure, Acute Liver) OR (Acute Hepatic Failure)) OR (Failure, Acute Hepatic)) OR (Acute Liver Failure)) OR (Hepatic Failure, Acute)) OR (Liver Failure, Fulminant)) OR (Fulminant Liver Failure)) OR (Fulminant Liver Failures)) OR (Fulminant Hepatic Failure)) OR (Fulminant Hepatic Failures)) OR (Fulminating Hepatic Failure)) OR (Fulminating Hepatic Failures)) OR (Hepatic Failure, Fulminating)) OR (Fulminating Liver Failure)) OR (Fulminating Liver Failures)) OR (Liver Failure, Fulminating)) OR (Hepatic Failure, Fulminant)
#6	#4 OR #5
#7	"Swine"[Mesh]
#8	((((((((((((Pigs) OR (Suidae)) OR (Warthogs)) OR (Phacochoerus)) OR (Wart Hogs)) OR (Hogs, Wart)) OR (Hog, Wart)) OR (Wart Hog)) OR (Pig)) OR (Porcine)) OR (Hog)) OR (Boar)) OR (Piglet)
#9	#7 OR #8
#10	"Dogs"[Mesh]
#11	((Dog) OR (Canis familiaris)) OR (Canine)
#12	#10 OR #11
#13	"Haplorhini"[Mesh]

#14	((((((Monkeys) OR (Monkey)) OR (Anthropoidea)) OR (Anthropoids)) OR (non-human primates)) OR (Macaque)) OR (Baboon)
#15	#13 OR #14
#16	"Sheep, Domestic"[Mesh]
#17	(((((((((Domestic Sheep) OR (Ovis aries)) OR (Ovis ammon aries)) OR (Mouflon)) OR (Mouflons)) OR (Ovis aries musimon)) OR (Ovis gmelini musimon)) OR (Sheep)) OR (Goats)) OR (Lamb)
#18	#16 OR #17
#19	"Rabbits"[Mesh]
#20	((((((((((((((((((((((((Rabbit) OR (Belgian Hare)) OR (Hare, Belgian)) OR (New Zealand Rabbits)) OR (Rabbit, New Zealand)) OR (Rabbits, New Zealand))
#21	#19 OR #20
#22	"Horses"[Mesh]
#23	((((((Horse) OR (Equus caballus)) OR (Horse, Domestic)) OR (Domestic Horse)) OR (Domestic Horses)) OR (Horses, Domestic)) OR (Equus przewalskii)
#24	#22 OR #23
#25	#9 OR #12 OR #15 OR #18 OR #21 OR #24
#26	#3 AND #6 AND #25

	Search Method (Web of Science)
#1	Mesenchymal stem cells (Topic) OR Stem Cell, Mesenchymal (Topic) OR Mesenchymal Stem Cell (Topic) OR Stem Cells, Mesenchymal (Topic) OR Mesenchymal Stromal Cells (Topic) OR Mesenchymal Stromal Cell (Topic) OR Stromal Cell, Mesenchymal (Topic) OR Stromal Cells, Mesenchymal (Topic) OR Wharton Jelly Cells (Topic) OR Wharton's Jelly Cells (Topic) OR Wharton's Jelly Cell (Topic) OR Whartons Jelly Cells (Topic) OR Bone Marrow Stromal Cells (Topic) OR Bone Marrow Stromal Cell (Topic) OR Bone Marrow Stromal Cells, Multipotent (Topic) OR Multipotent Bone Marrow Stromal Cell (Topic) OR Multipotent Bone Marrow Stromal Cells (Topic) OR Bone Marrow Stromal Stem Cells (Topic) OR Mesenchymal Progenitor Cell (Topic) OR Mesenchymal Progenitor Cells (Topic) OR Progenitor Cell, Mesenchymal (Topic) OR Progenitor Cells, Mesenchymal (Topic) OR Multipotent Mesenchymal Stromal Cells (Topic) OR Mesenchymal Stromal Cells, Multipotent (Topic) OR Multipotent Mesenchymal Stromal Cell (Topic) OR Bone Marrow Mesenchymal Stem Cells (Topic) OR Bone Marrow Mesenchymal Stem Cell (Topic) OR Adipose-Derived Mesenchymal Stem Cells (Topic) OR Adipose Derived Mesenchymal Stem Cells (Topic) OR Adipose-Derived Mesenchymal Stromal Cells (Topic) OR Adipose Derived Mesenchymal Stromal Cells (Topic) OR Mesenchymal Stem Cells, Adipose-Derived (Topic) OR Mesenchymal Stem Cells, Adipose Derived (Topic) OR Adipose Tissue-Derived Mesenchymal Stromal Cell (Topic) OR Adipose Tissue Derived Mesenchymal Stromal Cell (Topic) OR Adipose Tissue-Derived Mesenchymal Stromal Cells (Topic) OR Adipose Tissue Derived Mesenchymal Stromal Cells (Topic) OR Adipose Tissue-Derived Mesenchymal Stem Cell (Topic) OR Adipose Tissue Derived Mesenchymal Stem Cell (Topic) OR Adipose Tissue-Derived Mesenchymal Stem Cells (Topic) OR Adipose Tissue Derived Mesenchymal Stem Cells (Topic) OR Adipose-Derived Mesenchymal Stem Cell

	(Topic) OR Adipose Derived Mesenchymal Stem Cell (Topic) OR Umbilical Cord-derived Mesenchymal Stem Cells (Topic) OR Human Mesenchymal Stem Cells (Topic) OR Placenta-derived Mesenchymal Stem Cells (Topic)
#2	Liver Failure, Acute (Topic) OR Failure, Acute Liver (Topic) OR Acute Hepatic Failure (Topic) OR Failure, Acute Hepatic (Topic) OR Acute Liver Failure (Topic) OR Hepatic Failure, Acute (Topic) OR Liver Failure, Fulminant (Topic) OR Fulminant Liver Failure (Topic) OR Fulminant Liver Failures (Topic) OR Fulminant Hepatic Failure (Topic) OR Fulminant Hepatic Failures (Topic) OR Fulminating Hepatic Failure (Topic) OR Fulminating Hepatic Failures (Topic) OR Hepatic Failure, Fulminating (Topic) OR Fulminating Liver Failure (Topic) OR Fulminating Liver Failures (Topic) OR Liver Failure, Fulminating (Topic) OR Hepatic Failure, Fulminant (Topic)
#3	Swine (Topic) OR Pig (Topic) OR Pigs (Topic) OR Suidae (Topic) OR Warthogs (Topic) OR Phacochoerus (Topic) OR Wart Hogs (Topic) OR Hogs, Wart (Topic) OR Hog, Wart (Topic) OR Wart Hog (Topic) OR Porcine (Topic) OR Hog (Topic) OR Boar (Topic) OR Piglet (Topic)
#4	Dogs (Topic) OR Dog (Topic) OR Canis familiaris (Topic) OR Canine (Topic)
#5	Haplorhini (Topic) OR Monkeys (Topic) OR Monkey (Topic) OR Anthropoidea (Topic) OR Anthropoids (Topic) OR non-human primates (Topic) OR Macaque (Topic) OR Baboon (Topic)
#6	Sheep, Domestic (Topic) OR Domestic Sheep (Topic) OR Ovis aries (Topic) OR Ovis ammon aries (Topic) OR Mouflon (Topic) OR Mouflons (Topic) OR Ovis aries musimon (Topic) OR Ovis gmelini musimon (Topic) OR Sheep (Topic) OR Goats (Topic) OR Lamb (Topic)
#7	Rabbits (Topic) OR Rabbit (Topic) OR Belgian Hare (Topic) OR Hare, Belgian (Topic) OR New Zealand Rabbits (Topic) OR Rabbit, New Zealand (Topic) OR Rabbits, New Zealand (Topic) OR Zealand Rabbit, New (Topic) OR Zealand Rabbits, New (Topic) OR New Zealand White Rabbits (Topic) OR NZW Rabbits (Topic) OR NZW Rabbit (Topic) OR Rabbit, NZW (Topic) OR Rabbits, NZW (Topic) OR New Zealand Rabbit (Topic) OR New Zealand White Rabbit (Topic) OR Chinchilla Rabbits (Topic) OR Chinchilla Rabbit (Topic) OR Rabbit, Chinchilla (Topic) OR Rabbits, Chinchilla (Topic) OR Rabbit, Domestic (Topic) OR Domestic Rabbit (Topic) OR Domestic Rabbits (Topic) OR Rabbits, Domestic (Topic) OR Oryctolagus cuniculus (Topic) OR cuniculus, Oryctolagus (Topic)
#8	Horses (Topic) OR Horse (Topic) OR Equus caballus (Topic) OR Horse, Domestic (Topic) OR Domestic Horse (Topic) OR Domestic Horses (Topic) OR Horses, Domestic (Topic) OR Equus przewalskii (Topic)
#9	#3 OR #4 OR #5 OR #6 OR #7 OR #8
#10	#1 AND #2 AND #9

Search Method (Embase)	
#1	'mesenchymal stem cell'/exp
#2	'mesenchymal progenitor cell' OR 'stem cell, mesenchymal' OR 'mesenchymal stem cells' OR 'mesenchymal stromal cells' OR 'bone marrow-derived mesenchymal stem cells' OR 'adipose tissue-derived mesenchymal stem cells' OR 'umbilical cord-derived mesenchymal

	stem cells' OR 'adipose-derived mesenchymal stem cells' OR 'human mesenchymal stem cells' OR 'placenta-derived mesenchymal stem cells'
#3	#1 OR #2
#4	'acute liver failure'/exp
#5	'acute hepatic failure' OR 'liver failure, acute' OR 'fulminant hepatic failure'
#6	#4 OR #5
#7	'pig'/exp
#8	'boar' OR 'hog' OR 'porcine' OR 'sus (genus)' OR 'sus scrofa' OR 'swine' OR 'piglet'
#9	'dog'/exp
#10	'canis canis' OR 'canis domesticus' OR 'canis familiaris' OR 'canis lupus familiaris' OR 'dogs' OR 'canine'
#11	'haplorhini'/exp
#12	'haplorrhini' OR 'monkey' OR 'non-human primates' OR 'macaque' OR 'baboon'
#13	'domestic sheep'/exp
#14	'ovis ammon aries' OR 'ovis aries' OR 'ovis orientalis aries' OR 'ovis ovis' OR 'sheep (major species)' OR 'sheep, domestic' OR 'sheep' OR 'goats' OR 'lamb'
#15	'leporidae'/exp
#16	'leporid' OR 'leporids' OR 'rabbit' OR 'rabbits' OR 'rabbits and hares' OR 'cuniculus'
#17	'horse'/exp
#18	'equus (equus)' OR 'equus subg. equus' OR 'equus subgenus equus' OR 'horses'
#19	#7 OR #8
#20	#9 OR #10
#21	#11 OR #12
#22	#13 OR #14
#23	#15 OR #16
#24	#17 OR #18
#25	#19 OR #20 OR #21 OR #22 OR #23 OR #24
#26	#3 AND #6 AND #25

Search Method (Cochrane Library)	
#1	MeSH descriptor: [Mesenchymal Stem Cells] explode all trees
#2	（Stem Cell, Mesenchymal） OR （Stem Cells, Mesenchymal） OR （Mesenchymal Stem Cell） OR （Progenitor Cells, Mesenchymal） OR （Mesenchymal Progenitor Cells） OR （Mesenchymal Progenitor Cell） OR （Progenitor Cell, Mesenchymal） OR （Stromal Cell, Mesenchymal） OR （Stromal Cells, Mesenchymal） OR （Mesenchymal Stromal Cells） OR （Mesenchymal Stromal Cell） OR （Whartons Jelly Cells） OR （Wharton's Jelly Cells） OR （Wharton Jelly Cells） OR （Wharton's Jelly Cell） OR （Adipose Tissue-Derived Mesenchymal Stem Cells） OR （Mesenchymal Stem Cells, Adipose-Derived） OR （Adipose Tissue Derived Mesenchymal Stromal Cells） OR （Adipose Tissue Derived Mesenchymal Stem Cell） OR （Adipose Tissue Derived Mesenchymal Stromal Cell） OR （Adipose Tissue-Derived Mesenchymal Stromal Cell） OR （Adipose Tissue-Derived Mesenchymal Stromal Cells） OR （Adipose Derived Mesenchymal Stem Cells） OR （Adipose Derived Mesenchymal Stromal Cells） OR （Adipose-Derived Mesenchymal Stromal Cells） OR （Mesenchymal Stem Cells, Adipose

Derived）OR（Adipose-Derived Mesenchymal Stem Cell）OR（Adipose Tissue-Derived Mesenchymal Stem Cell） OR （Adipose Derived Mesenchymal Stem Cell） OR （Adipose-Derived Mesenchymal Stem Cells）OR（Adipose Tissue Derived Mesenchymal Stem Cells） OR （Multipotent Mesenchymal Stromal Cells） OR （Mesenchymal Stromal Cells, Multipotent） OR （Multipotent Mesenchymal Stromal Cell） OR （Bone Marrow Mesenchymal Stem Cell） OR （Bone Marrow Mesenchymal Stem Cells） OR （Bone Marrow Stromal Stem Cells） OR （Bone Marrow Stromal Cell） OR （Bone Marrow Stromal Cells） OR （Multipotent Bone Marrow Stromal Cells） OR （Multipotent Bone Marrow Stromal Cell） OR （Bone Marrow Stromal Cells, Multipotent） OR （Bone Marrow-derived Mesenchymal Stem Cells） OR （Umbilical Cord-derived Mesenchymal Stem Cells）OR（Human Mesenchymal Stem Cells）OR（Placenta-derived Mesenchymal Stem Cells）

#3 #1 OR #2

#4 MeSH descriptor: [Liver Failure, Acute] explode all trees

#5 （Hepatic Failure, Acute） OR （Failure, Acute Liver） OR （Acute Hepatic Failure） OR （Acute Liver Failure） OR （Failure, Acute Hepatic） OR （Hepatic Failure, Fulminating） OR （Fulminating Hepatic Failure） OR （Fulminating Liver Failure） OR （Liver Failure, Fulminant） OR （Fulminant Hepatic Failures） OR （Liver Failure, Fulminating） OR （Fulminant Hepatic Failure） OR （Fulminating Hepatic Failures） OR （Hepatic Failure, Fulminant） OR （Fulminant Liver Failures） OR （Fulminant Liver Failure） OR （Fulminating Liver Failures）

#6 #4 OR #5

#7 MeSH descriptor: [Swine] explode all trees

#8 （Suidae） OR （Pigs） OR （Wart Hog） OR （Warthogs） OR （Wart Hogs） OR （Phacochoerus） OR （Hog, Wart） OR （Hogs, Wart） OR （pig） OR （porcine） OR （boar） OR （piglet）

#9 MeSH descriptor: [Dogs] explode all trees

#10 （Canis familiaris） OR （Dog） OR （canine）

#11 MeSH descriptor: [Haplorhini] explode all trees

#12 （Monkeys） OR （Monkey） OR （Anthropoidea） OR （Anthropoids） OR （non-human primates） OR （Macaque） OR （Baboon）

#13 MeSH descriptor: [Sheep, Domestic] explode all trees

#14 （Domestic Sheep） OR （Ovis ammon aries） OR （Ovis aries） OR （Ovis aries musimon） OR （Mouflons） OR （Mouflon） OR （Ovis gmelini musimon） OR （sheep） OR （goats） OR （Lamb）

#15 MeSH descriptor: [Rabbits] explode all trees

#16 （Rabbit） OR （Hare, Belgian） OR （Belgian Hare） OR （Rabbits, Chinchilla） OR （Chinchilla Rabbit） OR （Chinchilla Rabbits） OR （Rabbit, Chinchilla） OR （cuniculus, Oryctolagus） OR （Rabbit, Domestic） OR （Domestic Rabbit） OR （Oryctolagus cuniculus） OR （Domestic Rabbits） OR （Rabbits, Domestic） OR （Rabbits, New Zealand） OR （NZW Rabbits） OR （Zealand Rabbit, New） OR （New Zealand Rabbits） OR （Rabbits, NZW） OR （Rabbit, New Zealand） OR （New Zealand Rabbit） OR （New Zealand White Rabbits） OR （NZW Rabbit） OR （New Zealand White Rabbit） OR （Zealand Rabbits, New） OR （Rabbit, NZW）

#17 MeSH descriptor: [Horses] explode all trees

#18 （Equus przewalskii） OR （Equus caballus） OR （Horse, Domestic） OR （Domestic

	Horse） OR （Horses, Domestic） OR （Horse） OR （Domestic Horses）
#19	#7 OR #8
#20	#9 OR #10
#21	#11 OR #12
#22	#13 OR #14
#23	#15 OR #16
#24	#17 OR #18
#25	#19 or #20 or #21 or #22 or #23 or #24
#26	#3 AND #6 AND #25
