# Supplementary material for: The Efficacy of Mesenchymal Stem Cell Therapy in Large Animal Models of Acute Liver Failure: A Meta-Analysis
Source: Int J Mol Sci. 2026 Mar 31;27(7):3175. doi: 10.3390/ijms27073175 (PMC13072887; doi:10.3390/ijms27073175)
Supplement: Supplementary file 1 [file ijms-27-03175-s001.zip › Supplementary File S2. Tables of subgroup and sensitivity analyses.pdf]

Supplementary File S2: Table S1. Results of subgroup analyses of the effect of MSCs therapy on survival rate.

Cell type	Time point	Heterogeneity		RR	95%-CI	*Z*	*p*-value
		I2	*p-value*				
BM-MSCs	**3 days**	**38%**	**0.20**	**1.45**	**[1.14, 1.84]**	**3.02**	**0.003**
	**5 days**	**0%**	**0.83**	**15.34**	**[4.02, 58.53]**	**4.00**	**<0.0001**
	**7 days**	**0%**	**0.91**	**22.33**	**[4.63, 107.72]**	**3.87**	**0.0001**
	**14 days**	**0%**	**1.00**	**27.00**	**[3.90, 186.92]**	**3.34**	**0.0008**
Non-BM-MSCs	3 days	86%	0.0007	2.03	[0.32, 12.74]	0.76	0.45
	5 days	0%	1.00	3.00	[0.80, 11.29]	1.62	0.10
	7 days	0%	0.71	3.93	[0.81, 19.19]	1.69	0.09
	14 days	0%	0.99	5.62	[0.80, 39.33]	1.74	0.08
**Administration route**							
Deep vein	3 days	65%	0.01	1.37	[0.94, 2.00]	1.62	0.10
	**5 days**	**0%**	**0.55**	**9.40**	**[3.77, 23.47]**	**4.80**	**<0.00001**
	**7 days**	**0%**	**0.57**	**12.29**	**[4.21, 35.90]**	**4.59**	**<0.00001**
	**14 days**	**0%**	**0.77**	**15.50**	**[4.12, 58.39]**	**4.05**	**<0.0001**
Peripheral vein	3 days	65%	0.06	0.98	[0.58, 1.66]	0.06	0.95
	5 days	0%	0.40	2.33	[0.40, 13.78]	0.94	0.35
**Dose**							
(0.7-2.5)×10^6/kg	3 days	75%	0.02	2.01	[0.51, 7.91]	1.00	0.32
	**5 days**	**0%**	**0.48**	**5.29**	**[1.08, 25.92]**	**2.06**	**0.04**
	**7 days**	**0%**	**0.37**	**5.86**	**[1.31, 26.14]**	**2.32**	**0.02**
(3.0-3.3)×10^6/kg	**3 days**	**0%**	**0.62**	**1.59**	**[1.19, 2.13]**	**3.14**	**0.002**
	**5 days**	**0%**	**0.63**	**18.33**	**[3.83, 87.84]**	**3.64**	**0.0003**
	**7 days**	**0%**	**1.00**	**27.00**	**[3.90, 186.92]**	**3.34**	**0.0008**
	**14 days**	**0%**	**1.00**	**27.00**	**[3.90, 186.92]**	**3.34**	**0.0008**
**Species**							
pig	3 days	71%	0.004	1.33	[0.89, 2.00]	1.39	0.17
	**5 days**	**0%**	**0.50**	**8.53**	**[3.42, 21.27]**	**4.60**	**<0.00001**
	**7 days**	**0%**	**0.55**	**11.86**	**[4.09, 34.43]**	**4.55**	**<0.00001**
	**14 days**	**0%**	**0.72**	**14.75**	**[3.97, 54.81]**	**4.02**	**<0.0001**
monkey	3 days	0%	0.33	1.13	[0.79, 1.62]	0.67	0.50
	5 days	76%	0.04	1.51	[0.22, 10.29]	0.42	0.68
	**7 days**	**0%**	**0.67**	**3.99**	**[1.25, 12.71]**	**2.34**	**0.02**
	**14 days**	**0%**	**0.32**	**6.14**	**[1.38, 27.27]**	**2.38**	**0.02**

Table S2. Results of sensitivity analyses of the effect of MSCs therapy on survival rate.

	Random-effects model				
**Survival rate at 3 days**	**RR 95%-CI**	***I2***	***p***	***Z***	**p-value**
Pooled estimate	1.24 [0.92, 1.67]	66%	0.004	1.41	0.16
Omitting Dongyan Shi 2017	1.17 [0.86, 1.59]	64%	0.01	0.97	0.33
Omitting Gang Guo 2019	1.31 [0.93, 1.86]	65%	0.009	1.53	0.13
Omitting Hongcui Cao 2012	1.34 [0.99, 1.83]	58%	0.03	1.87	0.06
Omitting Jian-Feng Sang 2016	1.30 [0.91, 1.88]	72%	0.002	1.42	0.15
Omitting Jun Li 2012	1.20 [0.85, 1.70]	68%	0.005	1.05	0.29
Omitting Niang-Cheng Lin 2019	1.19 [0.89, 1.59]	66%	0.007	1.18	0.24
Omitting Pan-Pan Cen 2019	1.20 [0.92, 1.57]	62%	0.01	1.33	0.18
Omitting Yuting Zeng 2024	1.24 [0.89, 1.73]	72%	0.002	1.28	0.20
**Survival rate at 5 days**	**RR 95%-CI**	***I2***	***p***	***Z***	**p-value**
Pooled estimate	4.86 [0.43, 55.38]	94%	<0.00001	1.27	0.20
Omitting Dongyan Shi 2017	4.09 [0.33, 50.03]	93%	<0.00001	1.10	0.27
**Omitting Gang Guo 2019**	**5.56 [2.38, 13.00]**	**0%**	**0.55**	**3.96**	<0.0001
Omitting Hongcui Cao 2012	5.34 [0.27, 106.53]	95%	<0.00001	1.10	0.27
Omitting Jian-Feng Sang 2016	4.40 [0.33, 59.51]	95%	<0.00001	1.12	0.26
Omitting Jun Li 2012 (a)	3.65 [0.40, 33.43]	92%	<0.00001	1.15	0.25
Omitting Niang-Cheng Lin 2019	5.34 [0.28, 103.43]	95%	<0.00001	1.11	0.27
Omitting Yuting Zeng 2024	5.36 [0.28, 102.84]	95%	<0.00001	1.11	0.27

Table S3. Results of subgroup analyses of the effect of MSCs therapy on ALT.

Cell type	Time point	Heterogeneity		SMD	95%-CI	*Z*	*p*-value
		I2	***p-value***				
BM-MSCs	**1 day**	**52%**	**0.08**	**-0.78**	**[-1.46, -0.10]**	**2.25**	**0.02**
	**3 days**	**0%**	**0.75**	**-0.47**	**[-0.92, -0.03]**	**2.09**	**0.04**
	5 days	0%	0.61	0.10	[-0.59, 0.78]	0.27	0.78
Non-BM-MSCs	1 day	0%	0.99	-0.47	[-1.41, 0.47]	0.99	0.32
	3 days	94%	<0.0001	-4.58	[-12.46, 3.31]	1.14	0.26
**Administration route**							
Deep vein	**1 day**	**0%**	**0.58**	**-0.67**	**[-1.11, -0.24]**	**3.05**	**0.002**
	**3 days**	**80%**	**0.0001**	**-1.70**	**[-2.95, -0.46]**	**2.68**	**0.007**
	5 days	0%	0.34	-0.04	[-0.82, 0.74]	0.11	0.92
Peripheral vein	1 day	60%	0.11	-0.52	[-1.66, 0.62]	0.89	0.37
	3 days	0%	0.88	-0.02	[-0.62, 0.59]	0.06	0.95

Dose							
(0.7-2.5)×10^6/kg	1 day	61%	0.08	-1.09	[-2.25, 0.07]	1.84	0.07
	3 days	0%	0.79	-0.62	[-1.33, 0.10]	1.69	0.09
(3.0-3.3)×10^6/kg	1 day	0%	0.90	-0.44	[-0.96, 0.09]	1.64	0.10
	3 days	0%	0.34	-0.53	[-1.12, 0.05]	1.78	0.07
(6.7-10)×10^6/kg	3 days	95%	<0.00001	-4.31	[-12.73, 4.11]	1.00	0.32
**Species**							
pig	**1 day**	**30%**	**0.20**	**-0.65**	**[-1.04, -0.26]**	**3.27**	**0.001**
	**3 days**	**76%**	**0.0008**	**-1.04**	**[-2.01, -0.08]**	**2.12**	**0.03**
	5 days	0%	0.61	0.10	[-0.59, 0.78]	0.27	0.78

Table S4. Results of sensitivity analyses of the effect of MSCs therapy on ALT.

	Random-effects model				
**ALT at 3 days**	**SMD 95%-CI**	***I2***	***p***	***Z***	**p-value**
Pooled estimate	-1.44 [-2.48, -0.41]	81%	<0.0001	2.73	0.006
**Omitting Hongcui Cao 2014**	**-0.91 [-1.63, -0.20]**	**63%**	**0.02**	**2.50**	**0.01**
Omitting Jian-Feng Sang 2016	-1.74 [-3.04, -0.43]	84%	<0.00001	2.61	0.009
Omitting jing jiang 2022	-1.51 [-2.68, -0.34]	84%	<0.00001	2.53	0.01
Omitting Jun Li 2012	-1.80 [-3.17, -0.44]	83%	<0.0001	2.59	0.009
Omitting Niang-Cheng Lin 2019	-1.65 [-2.89, -0.41]	84%	<0.00001	2.61	0.009
Omitting Xiao-Lei Shi 2013	-1.75 [-2.99, -0.52]	84%	<0.0001	2.78	0.005
Omitting Yuting Zeng 2024	-1.04 [-2.01, -0.08]	76%	0.0008	2.12	0.03

Table S5. Results of subgroup analyses of the effect of MSCs therapy on AST.

	**Time point**	**Heterogeneity**		**SMD**	**95%-CI**	***Z***	***p*-value**
**Cell type**		***I2***	***p-value***				
BM-MSCs	**1 day**	**0%**	**0.89**	**-0.78**	**[-1.31, -0.25]**	**2.87**	**0.004**
Non-BM-MSCs	1 day	0%	0.90	0.30	[-0.62, 1.23]	0.64	0.52
**Administration route**							
Deep vein	**1 day**	**26%**	**0.25**	**-0.58**	**[-1.08, -0.09]**	**2.31**	**0.02**
	3 days	94%	<0.0001	-1.00	[-5.84, 3.85]	0.40	0.69
**Dose**							
(0.7-2.5)×10^6/kg	1 day	3%	0.36	-0.18	[-0.83, 0.47]	0.53	0.59
	3 days	77%	0.04	0.50	[-1.14, 2.14]	0.60	0.55

(3.0-3.3)$\times10^{6}$/kg	**1 day**	**0%**	**0.74**	**-0.85**	**[-1.50, -0.19]**	**2.54**	**0.01**
**Species**							
pig	**1 day**	**5%**	**0.38**	**-0.51**	**[-0.97, -0.05]**	**2.17**	**0.03**
	3 days	77%	0.04	0.50	[-1.14, 2.14]	0.60	0.55

Table S6. Results of sensitivity analyses of the effect of MSCs therapy on AST.

	Random-effects model				
**AST at 3 days**	**SMD 95%-CI**	***I2***	***p***	***Z***	**p-value**
Pooled estimate	-0.37 [-2.23, 1.48]	85%	0.001	0.39	0.69
Omitting Jian-Feng Sang 2016	-0.47 [-4.20, 3.26]	93%	0.0002	0.25	0.81
Omitting Niang-Cheng Lin 2019	-1.24 [-3.31, 0.83]	81%	0.02	1.17	0.24
Omitting Yuting Zeng 2024（a）	0.50 [-1.14, 2.14]	77%	0.04	0.60	0.55

Table S7. Sensitivity analysis after excluding non-D-gal models.

**Survival rate**	**Time point**	**SMD 95%-CI**	***I2***	***p***	***Z***	**p-value**
Primary Analysis	3 days	1.25 [0.94, 1.67]	67%	0.003	1.53	0.13
	5 days	3.99 [0.81, 19.53]	93%	<0.00001	1.71	0.09
	7 days	7.33 [3.52, 15.29]	8%	0.37	5.31	<0.00001
	14 days	14.77 [4.53, 48.17]	0%	0.86	4.46	<0.00001
Sensitivity Analysis	3 days	1.26 [0.84, 1.89]	73%	0.005	1.10	0.27
	5 days	10.38 [3.63, 29.73]	0%	0.51	4.36	<0.0001
	7 days	16.83 [4.49, 63.12]	0%	0.82	4.19	<0.0001
	14 days	17.93 [3.95, 81.46]	0%	0.64	3.74	0.0002
**ALT**	**Time point**	**SMD 95%-CI**	***I2***	***p***	***Z***	**p-value**
Primary Analysis	1 day	-0.69 [-1.07, -0.32]	23%	0.25	3.66	0.0002
	3 days	-1.44 [-2.48, -0.41]	81%	<0.0001	2.73	0.006
	5 days	0.10 [-0.59, 0.78]	0%	0.61	0.27	0.78
Sensitivity Analysis	1 day	-0.68 [-1.09, -0.26]	41%	0.13	3.19	0.001
	3 days	-1.21 [-2.40, -0.01]	81%	0.0003	1.98	0.05
	5 days	0.10 [-0.59, 0.78]	0%	0.61	0.27	0.78
**AST**	**Time point**	**SMD 95%-CI**	***I2***	***p***	***Z***	**p-value**

Primary Analysis	1 day	-0.51 [-0.97, -0.05]	5%	0.38	2.17	0.03
	3 days	-0.37 [-2.23, 1.48]	85%	0.001	0.39	0.69
Sensitivity Analysis	1 day	-0.68 [-1.19, -0.18]	0%	0.67	2.64	0.008
	3 days	-0.27 [-1.17, 0.62]	-	-	0.60	0.55
