# Supplementary material for: The Efficacy of Mesenchymal Stem Cell Therapy in Large Animal Models of Acute Liver Failure: A Meta-Analysis
Source: Int J Mol Sci. 2026 Mar 31;27(7):3175. doi: 10.3390/ijms27073175 (PMC13072887; doi:10.3390/ijms27073175)
Supplement: Supplementary file 1 [file ijms-27-03175-s001.zip › Supplementary File S3. Publication bias.pdf]

Supplementary File S3. Publication bias

Survival rate at 3 days:

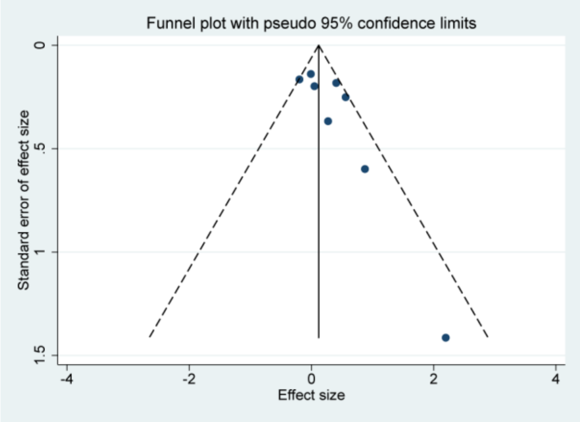

Egger's test: P=0.062

Survival rate at 5 days:

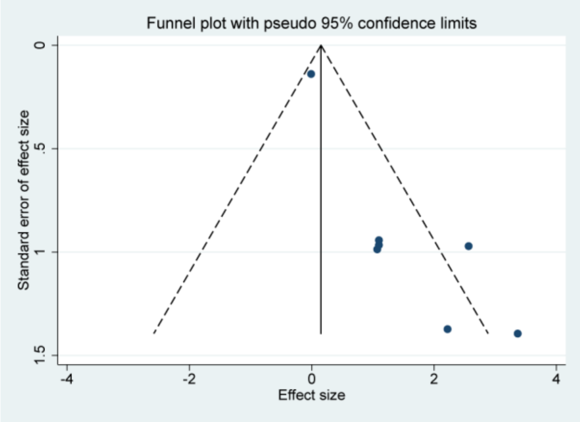

Egger's test: P=0.002

Survival rate at 7 days:

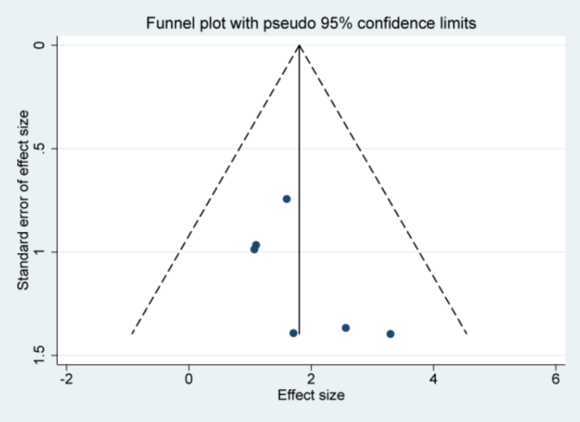

Egger's test: P=0.086

ALT at 1 day:

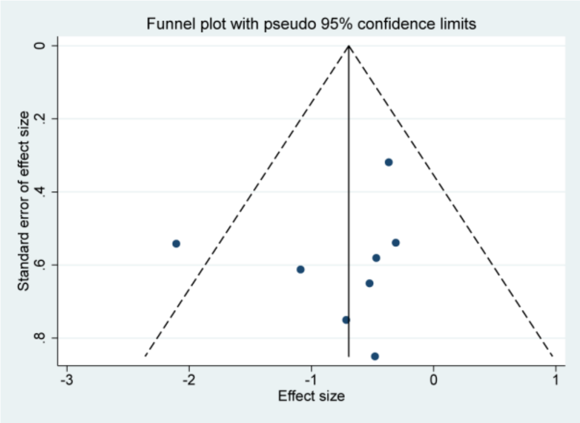

Egger's test: P=0.548

ALT at 3 days:

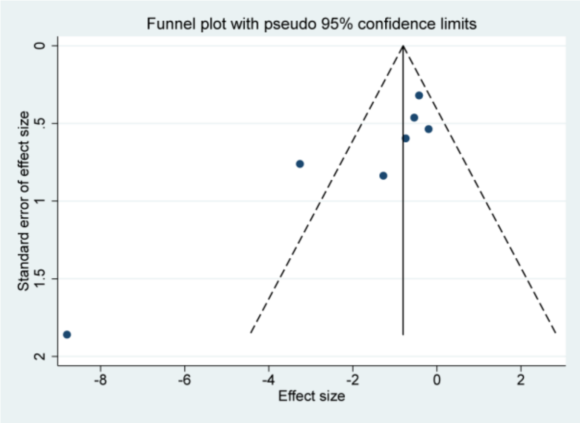

Egger's test: P=0.021
