# Supplementary material for: The Efficacy of Mesenchymal Stem Cell Therapy in Large Animal Models of Acute Liver Failure: A Meta-Analysis
Source: Int J Mol Sci. 2026 Mar 31;27(7):3175. doi: 10.3390/ijms27073175 (PMC13072887; doi:10.3390/ijms27073175)
Supplement: Supplementary file 1 [file ijms-27-03175-s001.zip › Supplementary File S4. Studies Characteristics and Outcomes.pdf]

Supplementary File S4: Table S1. Studies Characteristics and Survival Rate.

First author	Species	Model method	Source of MSCs	Admin route	Dose	Day 3	Day 5	Day 7	Day 14	P
Hongcui Cao 2012	Pig	1.5 g/kg D-Gal	hPMSCs; Xray-treated hPMSCs	Jugular venous catheter(JVC) or the portal vein(PV/PV-Xray)	1×10^8	66%(JVC)<br>100%(Control)<br><br>67%(PV-Xray)<br>100%(Control)<br><br>100%(PV)<br>100%(Control)	17%(JVC)<br>17%(Control)<br><br>50%(PV-Xray)<br>17%(Control)<br><br>83%(PV)<br>17%(Control)	17%(JVC)<br>0%(Control)<br><br>33%(PV-Xray)<br>0%(Control)<br><br>67%(PV)<br>0%(Control)	17%(JVC)<br>0%(Control)<br><br>33%(PV-Xray)<br>0%(Control)<br><br>67%(PV)<br>0%(Control)	P < 0.05 (PV group compared with the other three groups)
Jun Li 2012	Pig	1.5 g/kg D-Gal	hBMSCs	Intrahepatic portal vein(IPT); ear vein(PVT)	3×10^7	93%（PVT）<br>67%(Control)<br><br>100%（IPT）<br>67%(Control)	0%（PVT）<br>0%(Control)<br><br>93%（IPT）<br>0%(Control)	0%（PVT）<br>0%(Control)<br><br>87%（IPT）<br>0%(Control)	0%（PVT）<br>0%(Control)<br><br>87%（IPT）<br>0%(Control)	P < 0.05 (IPT group versus PVT and control groups)
Jian-Feng Sang 2016	Swine	0.3 g/kg D-Gal	pBM- MSCs	External ear vein(PV); portal vein(InP); arteria hepatica(AH) or intrahepatic(IH)	1×10^7	67%(PV)<br>84%(Control)<br><br>100%(InP)<br>84%(Control)<br><br>100%(AH)<br>84%(Control)<br><br>84%(IH)<br>84%(Control)	34%(PV)<br>0%(Control)<br><br>100%(InP)<br>0%(Control)<br><br>67%(AH)<br>0%(Control)<br><br>0%(IH)<br>0%(Control)	0%(PV)<br>0%(Control)<br><br>100%(InP)<br>0%(Control)<br><br>0%(AH)<br>0%(Control)<br><br>0%(IH)<br>0%(Control)	0%(PV)<br>0%(Control)<br><br>0%(InP)<br>0%(Control)<br><br>0%(AH)<br>0%(Control)<br><br>0%(IH)<br>0%(Control)	P<0.01 (InP group compared to the D-Gal group)

Supplementary File S4: Table S1. Studies Characteristics and Survival Rate.(continued)

First author	Species	Model method	Source of MSCs	Admin route	Dose	Day 3	Day 5	Day 7	Day 14	P
Dongyan Shi 2017	Pig	1.5 g/kg D-Gal	hBMSCs	Intrahepatic portal vein	3×10^6 /kg	94%(Group T) 53%(Group C)	87%(Group T) 6%(Group C)	87%(Group T) 0%(Group C)	87%(Group T) 0%(Group C)	NR
Pan-Pan Cen 2019	Pig	1.0 g/kg D-Gal	MenSCs	Portal vein	2.5×10^6 /kg	50%(Group T) 0%(Group C)	0%(Group T) 0%(Group C)	0%(Group T) 0%(Group C)	0%(Group T) 0%(Group C)	P < 0.001
Niang-Cheng Lin 2019	Pig	I-R Injury	Human adipose-derived MSCs	Splenic vein	2.4×10^7	80%(MSCs) 33%(Control)	50%(MSCs) 17%(Control)	50%(MSCs) 17%(Control)	40%(MSCs) 0%(Control)	P = 0.038
Gang Guo 2019	Monkey	α-amatoxin (25 μg/kg) and LPS (1 μg/kg)	hUC-MSCs	Peripheral infusion	1×10^7	100%(MSCs) 100%(Control)	100%(MSCs) 100%(Control)	100%(MSCs) 14%(Control)	100%(MSCs) 0%(Control)	NR
Yuting Zeng 2024	Monkey	40 μg/kg α-amanitin	hUC-MSCs	Peripheral infusion	1×10^7; 2×10^7	67%(1U MSC) 57%(Control) 83%(2U MSC) 57%(Control)	33%(1U MSC) 14%(Control) 50%(2U MSC) 14%(Control)	33%(1U MSC) 14%(Control) 50%(2U MSC) 14%(Control)	33%(1U MSC) 14%(Control) 50%(2U MSC) 14%(Control)	P < 0.001

NR: not reported.

Note: Significance symbols are based exclusively on original study reports.

Supplementary File S4: Table S2. Studies Characteristics and Serum ALT Levels.

First author	Species	Model method	Source of MSCs	Admin route	Dose	Day 1	Day 3	Day 5	Day 7	Day 14
Jun Li 2012	Pig	1.5 g/kg D-Gal	hBMSCs	Intrahepatic portal vein(IPT); ear vein(PVT)	3×10^7	↓(IPT) ↔(PVT)	↓(IPT) ↔(PVT)	↓(IPT) ↔(PVT)	↔(IPT) NA(PVT)	↔(IPT) NA(PVT)
Xiao-Lei shi 2013	Swine	0.3 g/kg D-Gal	pBM- MSCs	Portal vein	1×10^8	↔	↔	↔	↔	↔
Hongcui Cao 2014	Pig	1.5 g/kg D-Gal	hPMSCs	Intrahepatic portal vein	1×10^8	NR	↓	NA	NA	NA
Jiaojiao Xin 2015	Pig	1.5 g/kg D-Gal	hBMSCs	Intrahepatic portal vein	3×10^7	NR	NR	NR	NR	NR
Jian-Feng Sang 2016	Swine	0.3 g/kg D-Gal	pBM- MSCs	External ear vein(PV); portal vein(InP); arteria hepatica(AH) or intrahepatic(IH)	1×10^7	↔(PV) ↓(InP) ↓(AH) ↔(IH)	↔(PV) ↓(InP) ↓(AH) ↔(IH)	↔(PV) ↔(InP) ↔(AH) ↔(IH)	NR(PV, InP, AH, IH)	NR(PV, InP, AH, IH)
Pan-Pan Cen 2019	Pig	1.0 g/kg D-Gal	MenSCs	Portal vein	2.5×10^6/kg	↓	NA	NA	NA	NA

Niang-Cheng Lin 2019	Pig	I-R Injury	Human adipose-derived MSCs	Splenic vein	2.4×10^7	↔	↔	NA	NA	NA

Supplementary File S4: Table S2. Studies Characteristics and Serum ALT Levels.(continued)

First author	Species	Model method	Source of MSCs	Admin route	Dose	Day 1	Day 3	Day 5	Day 7	Day 14
Jing Jiang 2022	Pig	1.5 g/kg D-Gal	hBMSCs	Intrahepatic portal vein	3×10^6/kg	↔	↔	NA	NA	NA
Yuting Zeng 2024	Monkey	40 μg/kg α-amanitin	hUC-MSCs	Peripheral infusion	1×10^7; 2×10^7	↓(1-U MSC, 2-U MSC)	↓(1-U MSC, 2-U MSC)	NR(1-U MSC, 2-U MSC)	NR(1-U MSC, 2-U MSC)	NR(1-U MSC, 2-U MSC)

↑: significantly higher (P < 0.05); ↓: significantly lower (P < 0.05); ↔: no significant difference (P ≥0.05); NA: not assessed; NR: not reported..

Note: Significance symbols are based exclusively on original study reports.

Supplementary File S4: Table S3. Studies Characteristics and Serum AST Levels.

First author	Species	Model method	Source of MSCs	Admin route	Dose	Day 1	Day 3	Day 5	Day 7	Day 14
Jiaojiao Xin 2015	Pig	1.5 g/kg D-Gal	hBMSCs	Intrahepatic portal vein	3×10^7	NR	NR	NR	NR	NR
Jian-Feng Sang 2016	Swine	0.3 g/kg D-Gal	pBM- MSCs	External ear vein(PV); portal vein(InP); arteria hepatica(AH) or intrahepatic(IH)	1×10^7	↔(PV) ↔(InP) ↔(AH) ↔(IH)	↔(PV) ↓(InP) ↔(AH) ↔(IH)	↔(PV) ↔(InP) ↔(AH) ↔(IH)	NA(PV, InP, AH, IH)	NA(PV, InP, AH, IH)
Dongyan Shi 2017	Pig	1.5 g/kg D-Gal	hBMSCs	Intrahepatic portal vein	3×10^6/kg	↓	↔	↔	↔	↔
Pan-Pan Cen 2019	Pig	1.0 g/kg D-Gal	MenSCs	Portal vein	2.5×10^6/kg	↓	NA	NA	NA	NA
Niang-Cheng Lin 2019	Pig	I-R Injury	Human adipose-derived MSCs	Splenic vein	2.4×10^7	↔	↔	NA	NA	NA

Yuting Zeng 2024	Monkey	40 μg/kg α-amanitin	hUC-MSCs	Peripheral infusion	1×10^7; 2×10^7	↓(1-U MSC, 2-U MSC)	↓(1-U MSC, 2-U MSC)	NR(1-U MSC, 2-U MSC)	NR(1-U MSC, 2-U MSC)	NR(1-U MSC, 2-U MSC)

↑: significantly higher (P < 0.05); ↓: significantly lower (P < 0.05); ↔: no significant difference (P ≥0.05); NA: not assessed; NR: not reported..

Note: Significance symbols are based exclusively on original study reports.

Supplementary File S4: Table S4. Study Characteristics and IL-6 Levels.

First author	Species	Model method	Source of MSCs	Admin route	Dose	Day 1	Day 3	Day 5	Day 7	Day 14
Jiang-qiang Xiao 2013	Swine	0.3 g/kg D-Gal	pBM- MSCs	Portal vein	8×10^7	NR	↔	NR	↔	↔
Yuting Zeng 2024	Monkey	40 μg/kg α-amanitin	hUC-MSCs	Peripheral infusion	1×10^7; 2×10^7	↓(1-U MSC, 2-U MSC)	NR(1-U MSC, 2-U MSC)	NR(1-U MSC, 2-U MSC)	NR(1-U MSC, 2-U MSC)	NR(1-U MSC, 2-U MSC)

↑: significantly higher (P < 0.05); ↓: significantly lower (P < 0.05); ↔: no significant difference (P ≥0.05); NA: not assessed; NR: not reported.

Note: Significance symbols are based exclusively on original study reports.

Supplementary File S4: Table S5. Study Characteristics and TNF-α Levels.

First author	Species	Model method	Source of MSCs	Admin route	Dose	Day 1	Day 3	Day 5	Day 7	Day 14

Jiang-qiang Xiao 2013	Swine	0.3 g/kg D-Gal	pBM- MSCs	Portal vein	8×10^7	NR	↔	NR	↔	↔

↑: significantly higher ($P < 0.05$); ↓: significantly lower ($P < 0.05$); ↔: no significant difference ($P \geq 0.05$); NA: not assessed; NR: not reported.

Note: Significance symbols are based exclusively on original study reports.
